# Supplementary material for: Iterative improvement in the automatic modular design of robot swarms
Source: PeerJ Comput Sci. 2020 Dec 7;6:e322. doi: 10.7717/peerj-cs.322 (PMC7924708; doi:10.7717/peerj-cs.322)
Supplement: Supplemental Information 3 [file peerj-cs-06-322-s003.zip › argos3/doc/api/standalone/a00539_source.html]

ARGoS: plugins/robots/generic/simulator/quadrotor\_position\_default\_actuator.h Source File


- Main Page
- Related Pages
- Namespaces
- Classes
- Files

- File List
- File Members

# plugins/robots/generic/simulator/quadrotor\_position\_default\_actuator.h

Go to the documentation of this file.

```
00001 
00007 #ifndef QUADROTOR_POSITION_ACTUATOR_DEFAULT_H
00008 #define QUADROTOR_POSITION_ACTUATOR_DEFAULT_H
00009 
00010 #include <string>
00011 #include <map>
00012 
00013 namespace argos {
00014    class CQuadRotorPositionDefaultActuator;
00015 }
00016 
00017 #include <argos3/core/utility/math/rng.h>
00018 #include <argos3/core/simulator/actuator.h>
00019 #include <argos3/core/simulator/entity/composable_entity.h>
00020 #include <argos3/plugins/robots/generic/control_interface/ci_quadrotor_position_actuator.h>
00021 #include <argos3/plugins/simulator/entities/quadrotor_entity.h>
00022 
00023 namespace argos {
00024 
00025    class CQuadRotorPositionDefaultActuator : public CSimulatedActuator,
00026                                      public CCI_QuadRotorPositionActuator {
00027 
00028    public:
00029 
00030       CQuadRotorPositionDefaultActuator();
00031       virtual ~CQuadRotorPositionDefaultActuator() {}
00032 
00033       virtual void SetRobot(CComposableEntity& c_entity);
00034 
00035       virtual void Init(TConfigurationNode& t_tree);
00036 
00037       virtual void SetAbsolutePosition(const CVector3& c_pos);
00038       virtual void SetRelativePosition(const CVector3& c_pos);
00039       virtual void SetAbsoluteYaw(const CRadians& c_yaw);
00040       virtual void SetRelativeYaw(const CRadians& c_yaw);
00041 
00042       virtual void Update();
00043       virtual void Reset();
00044 
00045    protected:
00046 
00047       CQuadRotorEntity* m_pcQuadRotorEntity;
00048       CEmbodiedEntity* m_pcEmbodiedEntity;
00049       CQuadRotorEntity::SPositionControlData m_sDesiredPosData;
00050       
00051    };
00052 
00053 }
00054 
00055 #endif
```

---

Generated on 10 Jul 2018 for ARGoS by 
 1.6.1
